# An open-label, randomized controlled trial of sulfamethoxazole–trimethoprim for *Pneumocystis* prophylaxis: results of 52-week follow-up

**DOI:** 10.1093/rap/rkaa029

**Published:** 2020-07-06

**Authors:** Masako Utsunomiya, Hiroaki Dobashi, Toshio Odani, Kazuyoshi Saito, Naoto Yokogawa, Kenji Nagasaka, Kenchi Takenaka, Makoto Soejima, Takahiko Sugihara, Hiroyuki Hagiyama, Shinya Hirata, Kazuo Matsui, Yoshinori Nonomura, Masahiro Kondo, Fumihito Suzuki, Yasushi Nawata, Makoto Tomita, Mari Kihara, Waka Yokoyama-Kokuryo, Fumio Hirano, Hayato Yamazaki, Ryoko Sakai, Toshihiro Nanki, Ryuji Koike, Nobuyuki Miyasaka, Masayoshi Harigai

**Affiliations:** r1 Department of Rheumatic Diseases, Tokyo Metropolitan Tama Medical Center, Fuchu, Tokyo; r2 Departments of Pharmacovigilance; r3 Rheumatology, Graduate School of Medical and Dental Sciences, Tokyo Medical and Dental University (TMDU), Tokyo; r4 Department of Rheumatology, Musashino Red Cross Hospital, Musashino, Tokyo; r5 Division of Hematology, Rheumatology and Respiratory Medicine, Department of Internal Medicine, Faculty of Medicine, Kagawa University, Kida-gun, Kagawa; r6 Third Department of Internal Medicine, Obihiro-Kosei General Hospital, Obihiro, Hokkaido; r7 First Department of Internal Medicine, School of Medicine, University of Occupational and Environmental Health, Kitakyushu, Fukuoka; r8 Department of Rheumatology, Ome Municipal General Hospital, Ome, Tokyo; r9 Department of Medicine and Rheumatology, Tokyo Metropolitan Geriatric Hospital, Tokyo; r10 Department of Rheumatology, Yokohama City Minato Red Cross Hospital, Yokohama, Kanagawa; r11 Department of Hematology, Rheumatology, and Infectious Disease, Kumamoto University Graduate School of Medicine, Kumamoto, Kumamoto; r12 Department of Rheumatology, Kameda Medical Center, Kamogawa, Chiba; r13 Department of Rheumatology, Tokyo Kyosai Hospital, Tokyo; r14 Department of Rheumatology, Faculty of Medicine, Shimane University, Izumo, Shimane; r15 Department of Rheumatology, Soka Municipal Hospital, Soka, Saitama; r16 Center for Rheumatic Disease, Chibaken Saiseikai Narashino Hospital, Narashino, Chiba; r17 Clinical Research Center, Medical Hospital of Tokyo Medical and Dental University; r18 Division of Epidemiology and Pharmacoepidemiology, Institute of Rheumatology, Tokyo Women’s Medical University; r19 Department of Rheumatology, Tokyo Women’s Medical University School of Medicine, Tokyo, Japan

**Keywords:** *Pneumocystis* pneumonia, sulfamethoxazole–trimethoprim, prophylaxis, efficacy, safety, drug discontinuation rate, rheumatic disease, randomized controlled trial

## Abstract

**Objectives:**

The aim was to investigate the long-term prophylactic efficacy, drug retention and safety of low-dose sulfamethoxazole–trimethoprim (SMX/TMP) prophylaxis against *Pneumocystis* pneumonia (PCP).

**Methods:**

Adult patients with rheumatic diseases receiving prednisolone ≥0.6 mg/kg/day were randomized into the single-strength group (SS; SMX/TMP 400/80 mg daily), the half-strength group (HS; 200/40 mg daily) or the escalation group (ES; starting at 40/8 mg and increasing incrementally to 200/40 mg daily) and treated for 24 weeks, then observed for 52 weeks. The primary endpoint, the PCP non-incidence rate (non-IR) at week 24, has been reported previously. The secondary endpoints were the PCP non-IR at week 52, treatment discontinuation rate and adverse events.

**Results:**

Fifty-eight, 59 and 55 patients in the SS, HS and ES, respectively, received SMX/TMP. PCP did not develop in any of the patients by week 52. The estimated PCP non-IR in patients receiving SMX/TMP 200/40 mg daily (HS and ES) was 96.8–100%. Throughout the 52-week observation period, the overall discontinuation rate was significantly lower in HS than in SS (22.7 *vs* 47.2%, *P* = 0.004). The discontinuation rates attributable to adverse events were significantly lower in HS (19.1%, *P* = 0.007) and ES (20.3%, *P* = 0.007) than in SS (41.8%). The IRs of adverse events requiring SMX/TMP dose reduction before week 52 differed among the three groups, with a significantly higher IR in SS than in HS or ES (*P* = 0.007).

**Conclusion:**

SMX/TMP 200/40 mg had a high PCP prevention rate and was superior to SMX/TMP 400/80 mg in terms of drug retention and safety.

**Trial registration:**

University Hospital Medical Information Network Clinical Trials Registry, UMIN000007727.

**Key messages**

- Sulfamethoxazole–trimethoprim dosages for *Pneumocystis* pneumonia prophylaxis were compared in a randomized controlled trial.
- Sulfamethoxazole–trimethoprim 200/40 mg daily has good efficacy and tolerability in patients with rheumatic diseases.
- Sulfamethoxazole–trimethoprim 200/40 mg daily is recommended for *Pneumocystis* pneumonia prophylaxis in Japanese patients with rheumatic diseases.

## Introduction

*Pneumocystis* pneumonia (PCP) can have significant impacts on the clinical course of immunocompromized patients [1]. In particular, in non-HIV patients PCP can result in rapid deterioration and is more likely to have a poor prognosis [2] and should be prevented appropriately. Currently, sulfamethoxazole–trimethoprim (SMX/TMP) is widely used as the first-line drug for PCP prophylaxis [1, 3]. When used properly, the prevention rate is reportedly 89–100%. In a retrospective study of 1522 treatments of rheumatic diseases with high-dose CSs, the preventive efficacy of SMX/TMP was 93% [4]. In a study of RA patients using biologics, the PCP non-incidence rate (non-IR) was 100% in the prophylactic group and 98.4% in the non-prophylactic group [5]. However, many adverse events (AEs) related to this drug have been reported [3], which can necessitate switching to other drugs, discontinuation of prophylaxis and, eventually, development of PCP. Alternative drugs, such as pentamidine isethionate, atovaquone and dapsone, are considered to be less effective [6] and also produce various adverse drug reactions.

In PCP prophylaxis, striking a balance between benefit (i.e. prophylactic effect) and risk (i.e. AEs) is required. Guidelines for patients with HIV infection, haematological malignancies and solid organ transplantation indicate which patients are at risk for PCP and should receive prophylaxis [7–10]. Although PCP prophylaxis is indicated in some patients with rheumatic diseases [1, 5, 11–13], no clear consensus on prophylactic regimens has been developed because of the lack of robust evidence, and no official guidelines for PCP prophylaxis exist for patients with rheumatic diseases. To minimize the risks inherent in PCP prophylaxis, reduction of the AEs of SMX/TMP using an appropriate regimen is desirable. Prasad *et al*. [14] reported that SMX/TMP 400 mg/80 mg three times a week in renal transplant patients reduced AEs without affecting prophylaxis. Takenaka *et al*. [15] showed that SMX/TMP 400 mg/80 mg daily with dose escalation was superior to SMX/TMP 400 mg/80 mg daily in terms of the continuation rate in 41 patients with rheumatic diseases who started treatment with CSs. Suyama *et al*. [16] retrospectively compared SMX/TMP 400 mg/80 mg daily with 400 mg/80 mg in a dose escalation regimen in 59 patients with SLE and found that the latter was safer. In our previous report, using an open-label, randomized controlled trial, we compared the efficacy, drug retention and safety of three regimens (SMX/TMP 400 mg/80 mg daily, 200 mg/40 mg daily or 200 mg/40 mg with dose escalation) in 183 patients with systemic rheumatic diseases who started at a dosage of 0.6 mg/kg/day or more of prednisolone or an equivalent dosage of CS [17]. At week 24, the 200 mg/40 mg daily regimen was superior in efficacy, drug retention and safety. Herein, we report the results of observations at week 52 to verify long-term prophylactic effects and safety of the regimens.

## Methods

### Patients

The inclusion and exclusion criteria of the present study were described previously [17]. In brief, the inclusion criteria were follows: age ≥20 years; admission to one of the participating institutions for treatment of new-onset or relapsed systemic rheumatic disease in the study period; written informed consent; an oral prednisolone starting dosage at ≥0.6 mg/kg/day or an equivalent dosage of CS regardless of concomitant immunosuppressive drugs; no previous use of SMX/TMP, pentamidine isethionate or dapsone; and serum creatinine values within the upper limit of the normal range according to the institutional standard. Patients were excluded if they received a biologic agent, had a history of PCP or were unable to start SMX/TMP within 10 days of starting prednisolone.

### Study design

This study was an open-label, multicentre, randomized controlled trial, and the study design was described previously [17]. In brief, the patients were randomized into one of three arms at a 1:1:1 ratio by using computer-based, central, dynamic allocation with block randomization. Patients in the single-strength dosage group (SS) started SMX/TMP 400 mg/80 mg, the equivalent of a single-strength tablet, and patients in the half-strength dosage group (HS) started SMX/TMP 200 mg/40 mg, and both groups continued the same dosage for 24 weeks. Patients in the escalation group (ES) started SMX/TMP 40 mg/8 mg, the equivalent of 10% of a single-strength tablet, and the dosage was increased by 40 mg/8 mg weekly up to 200 mg/40 mg and continued for 24 weeks. All patients received SMX/TMP in granule form. After week 24 or discontinuation of the study, SMX/TMP use, including the dosage, interval and treatment duration, was left to the discretion of the attending physicians. The observation period was 52 weeks irrespective of continuation or discontinuation of the study regimen. This study was approved by the ethics committee of Tokyo Medical and Dental University Hospital (#2349) and the other participating institutions. This study was registered with the University Hospital Medical Information Network Clinical Trials Registry (UMIN000007727).

### Endpoints

PCP non-IRs at week 24 between SS and ES (the primary endpoint of this study) and the other comparisons between groups at week 24 have been reported previously [17]. The PCP non-IRs, treatment discontinuation rates and AEs at 52 weeks are reported here. PCP was diagnosed clinically by symptoms, laboratory tests and imaging by site investigators, with verification by the clinical event review committee, which included experts in pulmonary medicine, infectious diseases and rheumatology.

### Statistical analyses

The calculated sample size was 58 patients per group, assuming the PCP non-IR of SS to be 93% and that of ES to be 98%. The non-inferiority limit was set at 5%, one-sided α at 0.05, and β at 0.20 [15, 17]. For statistical analyses, treatment discontinuation rates were analysed using the Kaplan–Meier method and the log-rank test. Fisher’s exact test with adjusted residuals was used to compare the incidences of AEs. As a *post hoc* analysis, the PCP non-IRs of each group and the combined group of HS and ES were estimated using the Clopper–Pearson exact confidence interval [18] and the rule of three [19], because PCP developed in none of the patients.

## Results

### Randomization and follow-up

One hundred and eighty-three patients were randomized into one of three arms, with 58, 59 and 55 patients in SS, HS and ES, respectively, starting treatment with SMX/TMP. Twenty-nine, 43 and 34 patients in SS, HS and ES, respectively, continued the same regimen until week 52. Details are given in Supplementary Fig. S1, available at *Rheumatology Advances in Practice* online. Reasons for discontinuation of the regimens were AEs, prescription errors or the investigators’ discretion. All the patients were followed for 52 weeks, except those who died or were transferred to another hospital.

### Baseline characteristics of the patients

Supplementary Table S1, available at *Rheumatology Advances in Practice* online, shows the baseline characteristics of the patients. The mean age, percentage of female patients and mean body weight were similar across the groups. There were no significant differences in terms of a background of rheumatic diseases, co-morbidities or treatments before enrolment. The range of prednisolone-equivalent CS dosages was 0.94–0.97 mg/kg/day at baseline, 10–12.5 mg/day at week 24, and 7–8 mg/day at week 52. The proportion of the patients receiving i.v. pulsed methylprednisolone between weeks 0 and 12 was 20–32.2%. The proportion of those receiving an immunosuppressant was 67.8–81.8, 65.5–78.2, 63.8–71.2 and 65.5–74.6%, at weeks 0–12, 12–24, 24–36 and 36–52, respectively.

### Efficacy and drug discontinuation rate

**Fig. 1:**
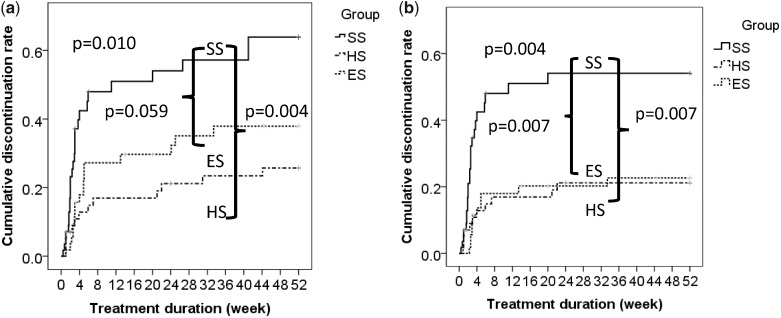
Discontinuation rates of the allocated treatments by Kaplain–Meier analysis The discontinuation rates of the allocated treatments attributable to any reasons (**a**) and attributable to adverse events (**b**) are shown. Cumulative treatment discontinuation rates were compared using the log-rank test among groups. ES: escalation group; HS: half-strength dosage group; SS: single-strength dosage group.

No PCP cases were reported up to week 52, and the PCP non-IR was estimated by using the Clopper–Pearson exact confidence interval in *post hoc* analysis [18]. The estimated non-IR at week 52 in SS, HS and ES was 93.8–100, 93.9–100 and 93.5–100%, respectively. Given that the patients in HS and ES received 200 mg/40 mg SMX/TMP daily for 52 and 47 weeks, respectively, we combined these two groups and estimated the PCP non-IR of the combined group to be 96.8–100% (*n* = 114). Estimation using the rule of three showed almost the same results [19]. The cumulative discontinuation rates due to any reason are shown in Fig. 1a using the Kaplan–Meier curves. HS showed a significantly lower cumulative discontinuation rate than SS (47.2 *vs* 22.7%, *P* = 0.004), which also tended to be lower in ES (31.6%) than in SS (*P* = 0.059). Fig. 1b shows the cumulative discontinuation rates attributable to AEs. A significant difference was observed between SS (41.8%) and ES (20.3%) (*P* = 0.007) and between SS and HS (19.1%) (*P* = 0.007).

### Safety

Table 1 summarizes the AEs and their breakdown during the study period. Although no significant difference in the overall IRs of AEs and serious AEs was observed, there was a significant difference in the proportion of patients with AEs who required SMX/TMP dosage reduction (*P* = 0.007) and of patients with AEs of special interest (*P* = 0.001) across all three groups, with SS showing the highest proportion. Among the AEs of special interest, thrombocytopenia and hyponatraemia were observed more frequently in SS, but the difference was not tested statistically owing to the relatively small number of cases.

**Table 1:** Adverse events reported by week 52

Adverse event	SS (*n* = 58)	HS (*n* = 59)	ES (*n* = 55)	***P*-value** ^a^
AE, *n* (%)	34 (58.6)	25 (42.4)	26 (47.3)	0.201
Serious AE^b^, *n* (%)	9 (15.5)	12(20.3)	6 (10.9)	0.408
AE requiring SMX/TMP dose reduction, *n* (%)	12 (20.7)	2 (3.4)^c^	4 (7.3)^c^	0.007
AE requiring SMX/TMP discontinuation, *n* (%)	12 (20.7)	8 (13.6)	6 (10.9)	0.330
AE leading to death, *n* (%)	1 (1.7)	3 (5.1)	1 (1.8)	0.622
AE of special interest, *n* (%)	25 (43.1)	10 (16.9)^c^	9 (16.4)^c^	0.001
Fever, *n* (%)	2 (3.4)	0 (0)	0 (0)	ND
Rash, *n* (%)	5 (8.6)	2 (3.4)	1 (1.8)	ND
Appetite loss, *n* (%)	1 (1.7)	0 (0)	1 (1.8)	ND
Anaemia, *n* (%)	1 (1.7)	1 (1.7)	0 (0)	ND
Leucocytopenia, *n* (%)	1 (1.7)	1 (1.7)	0 (0)	ND
Thrombocytopenia, *n* (%)	9 (15.5)	3 (5.1)	4 (7.3)	ND
Elevated LFT, *n* (%)	6 (10.3)	4 (6.8)	4 (7.3)	ND
Elevated serum creatinine, *n* (%)	3 (5.2)	0 (0)	1 (1.8)	ND
Hyponatraemia, *n* (%)	6 (10.3)	1 (1.7)	0 (0)	ND
Hyperpotassaemia, *n* (%)	3 (5.2)	1 (1.7)	1 (1.8)	ND

Adverse events (AEs) reported in each group by week 52 were shown. Neither the incidence rates of overall AEs and serious AEs nor the rate of AEs requiring SMX/TMP dose reduction and AEs of special interest differed significantly among the three groups.

a By Fisher’s exact test.

b Serious AE: sepsis, organizing pneumonia, severe liver failure, flare of rheumatic disease, rash requiring hospitalization, thrombocytopenia requiring hospitalization, mental disorder requiring hospitalization or AE resulting in death.

c *P* < 0.05 by adjusted residues *vs* SS.

AE: adverse event; ES: escalation group; HS: half-strength dosage group; LFT: liver function test; ND: not detected; SMX/TMP: sulfamethoxazole–trimethoprim; SS: single-strength dosage group.

## Discussion

In the present study, PCP did not develop in any of the patients in the 52-week observation period. The accumulated overall discontinuation rate for 52 weeks was significantly lower in HS than in SS, suggesting that the half-strength regimen had the better benefit–risk balance. A number of reasons might account for the lack of development of PCP in the present study. The physicians in charge at the participating institutions were proficient in treating rheumatic diseases and were knowledgeable about PCP prophylaxis. Many patients continued PCP prophylaxis even after stopping the allocated treatment with SMX/TMP; the numbers of patients without prophylaxis were 6 in weeks 0–12, 10 in weeks 12–24, and 17 in weeks 24–52. The lack of development of PCP in this study is understandable in view of the fact that the incidence of PCP without prophylaxis is reportedly 2.3–8.97% [5] and that the median prednisolone dosage administered at week 24 in this study was <15 mg/day, at which discontinuation of prophylaxis may be considered safely [4].

Some differences were observed between HS and ES in the drug discontinuation rates. The cumulative discontinuation rates by AEs did not differ significantly between HS and ES; the observed AEs, such as thrombocytopenia, elevated liver function test and hyponatraemia, were mostly dose dependent, with the exception of rash. This breakdown of AEs is consistent with previous reports [15, 16]. In contrast, HS had a lower drug discontinuation rate for all reasons than ES, mainly resulting from prescription errors in ES. In patients with allergy to multiple drugs, SMX/TMP may be administered under the escalation regimen. Recently, Suyama *et al*. [16] reported that patients with SLE, especially those with positive anti-Ro/SSA antibodies, had a higher incidence of adverse drug reactions to SMX/TMP. If true, the escalation regimen might be a better treatment option for these patients.

This study has some limitations. First, there was a detection bias attributable to nonblinding. The investigators might have expected more AEs in SS. Second, the efficacy and safety of a follow-up period >52 weeks are unknown. However, the median time from the start of treatment with CS to the onset of PCP was reportedly 12 weeks [20], and a 52-week observation period appeared to be sufficient in this context. Third, patients with reduced renal function and patients with low body weight were excluded. In addition, >60% of the patients were female; the mean body weight of the patients was <60 kg in all three groups, and only five patients weighed >80 kg. Fourth, not a large number of patients were enrolled in this study. Fifth, we did not test an alternate-day regimen in this study. This could be a challenge for the future.

## Conclusion

In patients with the characteristics included in the present study, SMX/TMP dosage reduction decreased the cumulative drug discontinuation rate and AEs requiring SMX/TMP dosage reduction. In patients with normal serum creatinine concentrations and similar body weights to those enrolled in the present study, SMX/TMP 200 mg/40 mg might provide a favourable benefit–risk balance in PCP prophylaxis.

## Supplementary Material

rkaa029_Supplementary_Data
