# Supplementary material for: An open-label, randomized controlled trial of sulfamethoxazole–trimethoprim for Pneumocystis prophylaxis: results of 52-week follow-up
Source: Rheumatol Adv Pract. 2020 Jul 6;4(2):rkaa029. doi: 10.1093/rap/rkaa029 (PMC7585401; doi:10.1093/rap/rkaa029)
Supplement: rkaa029_Supplementary_Data [file rkaa029_supplementary_data.zip › ST-RCT_52w_supplementary_file_for_submission final.docx]

**SUPPLEMENTARY MATERIAL**

**Supplementary Table S1 Patient Characteristics**

|  | | SS  (n = 58) | HS  (n = 59) | ES  (n = 55) |
| --- | --- | --- | --- | --- |
| Age, years | | 58.5 ± 15.0 | 58.1 ± 15.9 | 60.1 ± 14.4 |
| Female, % | | 63.8 | 64.4 | 70.9 |
| Body weight, kg | | 55.9 ± 11.8 | 56.8 ± 10.9 | 54.5 ± 9.9 |
| Diagnosis | |  |  |  |
|  | RA, % | 8.6 | 6.8 | 7.3 |
|  | SLE, % | 10.3 | 11.9 | 10.9 |
|  | PM/DM, % | 19.0 | 37.3 | 29.1 |
|  | Vasculitis syndrome, % | 44.8 | 25.4 | 30.9 |
|  | Others ^a^, % | 17.2 | 18.6 | 21.8 |
| Disease duration, months [IQR] | | 2 [1–5] | 3 [2–7] | 4 [2–9] |
| Comorbidities, % | | 72.4 | 79.7 | 78.2 |
|  | ILD, % | 38.0 | 44.1 | 43.6 |
|  | Other lung comorbidities ^b^, % | 12.1 | 8.5 | 5.5 |
|  | Hypertension, % | 13.8 | 18.6 | 14.5 |
|  | Diabetes, % | 6.9 | 5.1 | 14.5 |
|  | CVD ^c^, % | 3.4 | 5.1 | 5.5 |
|  | CKD, % | 1.7 | 0 | 0 |
|  | Malignancies, % | 6.9 | 11.9 | 9.1 |
|  | Others, % | 41.4 | 42.3 | 36.4 |
| Baseline laboratory data | |  |  |  |
|  | WBC, /μL  (NR, 3300 - 8600) | 10401 ± 5359 | 9901 ± 4767 | 9743 ± 5177 |
|  | Lymphocytes, /μL | 1766 ± 1106 | 1933 ± 1244 | 1656 ± 877 |
|  | IgG, mg/dL  (NR, 861 - 1747) | 1676 ± 677 | 1668 ± 679 | 2006 ± 1945 |
| Treatment before enrollment ^d^ | |  |  |  |
|  | CS, % | 15.5 | 13.3 | 14.5 |
|  | Dosage of CS ^e^, mg/day [IQR] | 13.8 [5–15] | 8.8 [5–10.6] | 6.8 [5–8.125] |
|  | IS ^f^, % | 1.7 | 3.2 | 7.3 |
|  | Biologics, % | 1.7 | 0 | 0 |
| Dosage of concomitant corticosteroid | |  |  |  |
|  | At baseline, mg/kg/day [IQR] | 0.97 [0.89–1.01] | 0.97 [0.81–1.02] | 0.94 [0.75–1.05] |
|  | At week 24, mg/day [IQR] | 12.5 [10–14.25] | 11 [9–15] | 10 [9–12.5] |
|  | At week 52, mg/day [IQR] | 8[5.25-10] | 7[6-8] | 7.5[5-10] |
| Other immunosuppressive treatment  between weeks 0–12 | |  |  |  |
|  | IV pulsed mPSL, % | 20.6 | 32.2 | 20 |
|  | IS, % | 70.6 | 67.8 | 81.8 |
|  | Biologics, % | 1.7 | 3.4 | 1.8 |
| Other immunosuppressive treatment  between weeks 12–24 | |  |  |  |
|  | IV pulsed mPSL, % | 1.7 | 3.4 | 0 |
|  | IS, % | 65.5 | 72.9 | 78.2 |
|  | Biologics, % | 1.7 | 1.7 | 3.6 |
| Other immunosuppressive treatment  between weeks 24-36 | |  |  |  |
|  | IV pulsed mPSL, % | 0 | 0 | 0 |
|  | IS, % | 63.8 | 71.2 | 70.9 |
|  | Biologics, % | 1.7 | 1.7 | 3.6 |
| Other immunosuppressive treatment  between weeks 36-52 | |  |  |  |
|  | IV pulsed mPSL, % | 0 | 0 | 0 |
|  | IS, % | 65.5 | 74.6 | 72.7 |
|  | Biologics, % | 5.2 | 3.4 | 7.3 |

Values conforming to the normal distribution were expressed as the mean ± SD. Values not conforming to the normal distribution were expressed as the median [interquartile range].

SS: the single-strength group; HS: the half-strength group; ES: the escalation group; RA: rheumatoid arthritis; SLE: systemic lupus erythematosus; PM: polymyositis; DM: dermatomyositis; IQR: interquartile range; ILD: interstitial lung disease; CVD: cardiovascular disease; CKD: chronic kidney disease; WBC: white blood cell; NR: normal range; CS: corticosteroid; mPSL: methylprednisolone; IS: immunosuppressive drug; IV: intravenous

^a^ Others include systemic sclerosis, mixed connective tissue diseases, Sjogren’s syndrome, adult-onset Still’s disease, relapsing polychondritis, IgG4-related disease, and antiphospholipid syndrome.

^b^ Other lung comorbidities include chronic obstructive lung disease, bronchiectasis, bronchial asthma, pulmonary hypertension, and previous pulmonary tuberculosis.

^c^ Cardiovascular diseases include cerebral infarction, cerebral hemorrhage, myocardial infarction, and angina pectoris.

^d^ Treatment given for 84 days before enrollment (i.e., starting or intensifying immunosuppressive treatment).

^e^ Prednisolone equivalent dose.

^f^ Immunosuppressive drugs include azathioprine, cyclophosphamide, cyclosporine, methotrexate, mizoribine, and mycophenolate mofetil and tacrolimus.

**Supplementary figure legend**

**Supplementary figure S1. Randomization and follow up.** One hundred eighty-three patients were randomized into one of the three arms, with 58, 59, and 55 patients in SS, HS, and ES, respectively, starting treatment with SMX/TMP. Between weeks 24 and 52, three, three, and one patients in SS, HS, and ES, respectively, discontinued this study, and three, two, and three patients in the respective groups stopped or reduced SMX/TMP due to the physician’s decision or AE.

SS: the single-strength dosage group; HS: the half-strength dosage group; ES: the escalation group, D/C: discontinued; PP: per protocol
