# Supplementary figures and images for: An open-label, randomized controlled trial of sulfamethoxazole–trimethoprim for Pneumocystis prophylaxis: results of 52-week follow-up
Source: Rheumatol Adv Pract. 2020 Jul 6;4(2):rkaa029. doi: 10.1093/rap/rkaa029 (PMC7585401; doi:10.1093/rap/rkaa029)

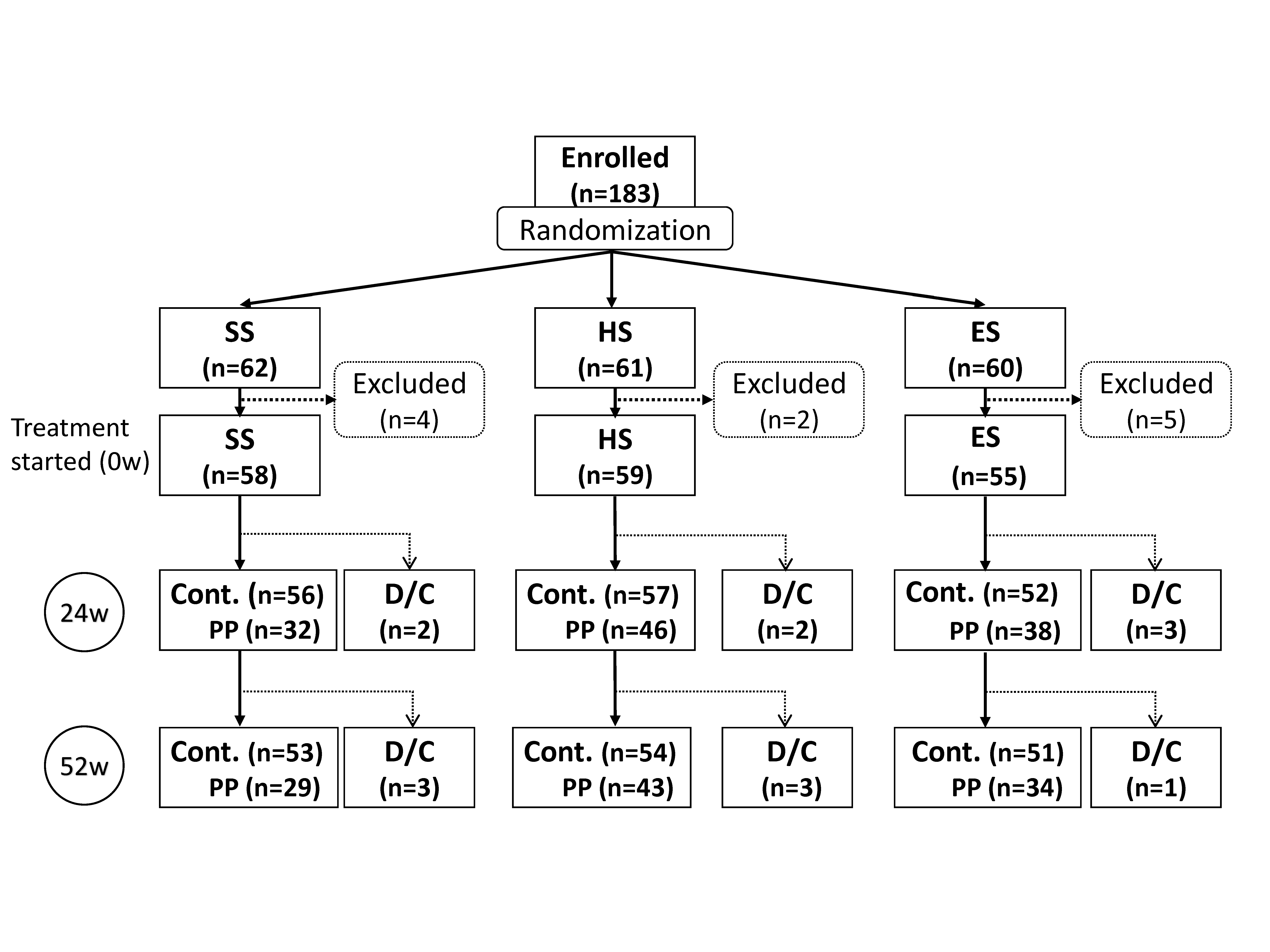

Supplement: rkaa029_Supplementary_Data [file rkaa029_supplementary_data.zip › Supplementary Figure S1.tif]
